# Continuously tuneable single electrode pair liquid crystal optical vortex generators

**DOI:** 10.1515/nanoph-2024-0047

**Published:** 2024-04-19

**Authors:** Camron Nourshargh, Alec Xu, Patrick S. Salter, Martin J. Booth, Steve J. Elston, Stephen M. Morris

**Affiliations:** Department of Engineering Science, 6396 University of Oxford, Oxford, UK

**Keywords:** optical vortex beams, liquid crystal devices, multi-order, two-photon polymerization, direct laser writing

## Abstract

In this work, we demonstrate the use of two-photon polymerization direct laser writing in the production of continuously tuneable optical vortex beam (OV) generators in a liquid crystal (LC) layer sandwiched between glass substrates. Results are presented that show how an OV generator can be inscribed into a 20 μm-thick LC layer and how the order of the OV beam can be tuned with the application of a voltage. Importantly, only a single pair of electrodes is needed to tune the order of the vortex as the required phase profile is generated through the 3D structuring of the polymer network using the laser writing process. Following the design and fabrication of the LC-OV generator, a Mach–Zehnder interferometer is subsequently employed, in conjunction with polarizing optical microscopy, to characterize the devices to confirm the generation of OVs of different orders and to determine the corresponding chirality. The paper concludes by considering whether these LC-OV generators can function at a range of different operation wavelengths. Such devices would be of potential importance in applications ranging from optical communications to quantum physics.

## 1 Introduction

Optical vortices (OVs), or vortex beams, have received a lot of attention since they were first proposed in 1989 [1], [2]. OVs have a helicoidal phase profile, resulting in a phase singularity along the optical axis, which, in the paraxial regime, gives rise to a zero in the intensity at the center of the OV. As a result of their helicoidal profile, OVs carry orbital angular momentum (OAM). Unlike spin angular momentum, which is carried by circularly polarized light and is limited to values of ±*ℏ* per photon, the angular momentum of an OV can take the value *lℏ*, where *l* describes both the chirality and the topological charge of the OV [3]. Controlling the angular momentum that OV beams carry makes them highly desirable in a number of different fields, such as atomic physics [4], where they can be used to excite high angular momentum transitions, and optical tweezing [5], where they can be used to rotate particles that are optically trapped. OVs can also be used in classical and quantum communications by way of modal division multiplexing [6], [7], [8]. In such applications, each separate vortex mode can be used as a single channel in a modulation scheme that can be subsequently multiplexed, transmitted, and separated at the receiver, thus reducing the required bandwidth in the classical case [9] or yielding a higher dimensionality of quantum key distribution in the quantum case [10].

There are two principal mechanisms through which OVs are typically generated: geometric phase and linear phase transformations. Geometric phase shifts, also known as Pancharatnam–Berry (PB) phase shifts, are, for example, those imparted on circularly polarized light as it passes through a half-wave plate. Here, the chirality of the polarization is inverted, and the phase of the subsequent light is determined by the orientation of the fast axis of the waveplate. By patterning the orientation of the fast axis, the phase profile of a circularly polarized beam can be modified into an optical vortex as has been demonstrated using a q-plate [11], [12]. As they rely on a half-wave retardance, q-plates must be developed in anisotropic media, for example, metasurfaces [13] and liquid crystal (LC) devices [14], [15].

While both approaches have yielded functional vortex beam generators, with some tuneability over wavelength, the fast axis must be patterned for the desired vortex order: for an *n*th order vortex, the fast axis must rotate by *nπ* around the optic axis of the device [16], [17]. As such they offer no OAM tuneability. In this work, we demonstrate an approach whereby the order of the OAM can be tuned.

Linear phase transforms are induced by spatially varying the optical path length through which the beam propagates. The simplest implementation of this is in helicoidal phase plates (HPPs), or spiral phase plates, as they are commonly known [18]. An HPP is generally a fixed refractive element that is designed to produce a desired vortex order at a given wavelength, but these tend to only be tuned by mechanical deformation rather than through the application of an electrical stimulus.

LC spatial light modulators (SLMs) can be used to generate OVs from linearly polarized light [19], [20]. The high degree of tuneability they provide, due to their pixelated LC on silicon design, gives them the ability to generate OVs up to extremely high orders and over the entire visible spectrum. The main drawback of SLMs, and the reason they are not used widely for OV generation, is their cost, which is largely attributed to the complex circuitry required for the high pixel density on the silicon backplane. However, approaches for simplifying the driving electronics of an LC device for OV generation have been demonstrated in recent years. For example, in [21], [22], two-photon laser ablation was used to pattern the electrodes in an LC glass device. By segmenting the electrodes into 72 individually addressable electrodes, the authors were able to produce nondiffracting OVs with orders ranging from −36 to +36.

**Figure 1:**
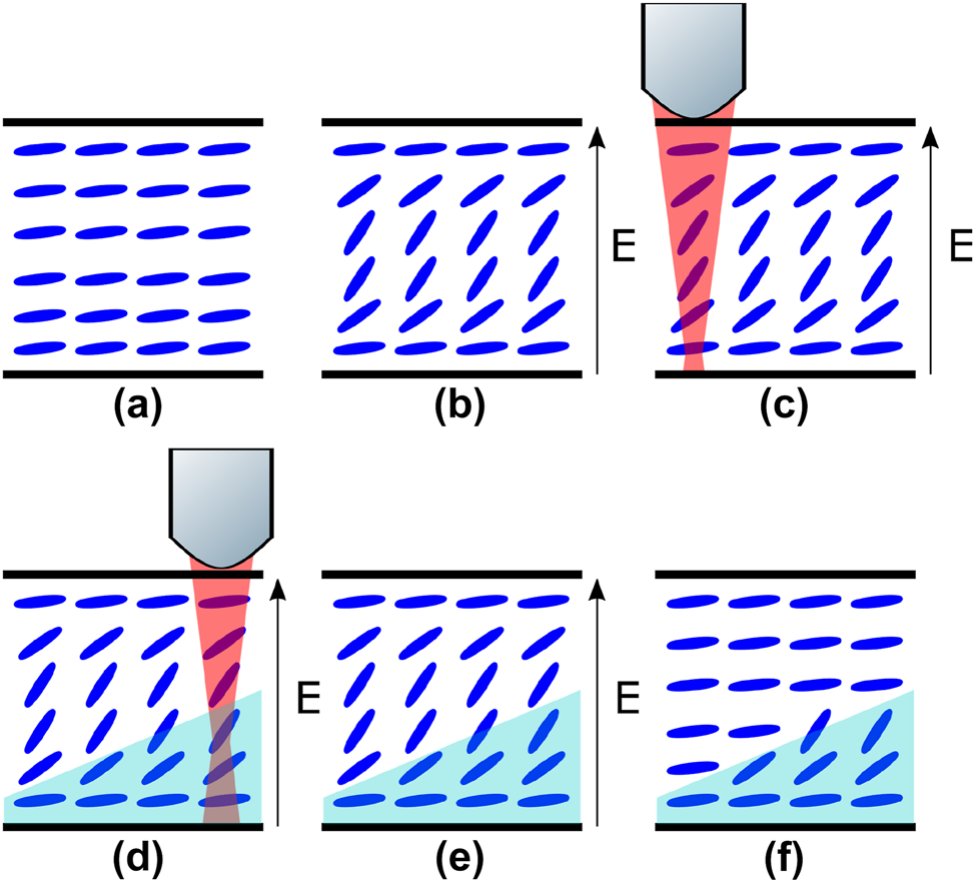
Illustration of the laser fabrication process used to write an optical vortex beam generator into a liquid crystal device. (a) No voltage applied, (b) voltage applied to reorient the LC director, (c) formation of a polymer network at a location defined by the focal volume of the laser writer, (d) translation of the device relative to the position of the laser focus, (e) polymerized region (shown as the shaded light blue region), and (f) director alignment locked in place by the polymer network while the remaining nonpolymerized region has relaxed back to the initial planar alignment.

In this work, we demonstrate OV beam generation using a laser written photopolymerized LC layer sandwiched between glass substrates with a single electrode pair. Specifically, the paper describes the development of a laser-written LC device that can be tuned in both OV order and operating wavelength with a single applied voltage. Liquid crystalline polymer networks have already shown great promise in the field of light control [23], [24]. Two-photon polymerization was employed to locally fix the director orientation (the average pointing direction of the LC molecules) enabling the patterning of the desired 2- or 3-D phase profile in the LC layer [25], [26]. After the fabrication process, the polymerized regions remain fixed while the nonpolymerized regions can be addressed electrically, enabling a reorientation of the director as illustrated in Figure 1.

For the OV generator, a helicoidal phase plate was written into the LC when a high voltage was applied to the LC layer in order to obtain a homeotropic alignment of the director. The benefit of writing in the presence of a voltage is that it results in the maximum vortex order possible when no voltage was applied to the device postfabrication.

The resultant device can be understood as two devices: the laser written polymer structure, with a fixed refractive index, *n*_*o*_ (the ordinary refractive index of the LC mixture), and the secondary structure, with a conjugate shape and a voltage controllable refractive index, which in the off-state is equivalent to the average refractive index of the LC mixture, *n*_ave_. In this way, we can calculate the linear phase imparted on light polarized along the rubbing direction as:

(1) $$\Phi \left(x,y\right)=\frac{2\pi n_{o}h\left(x,y\right)}{\lambda_{0}}+\frac{2\pi n_{\mathrm{ave}}\left(L-h\left(x,y\right)\right)}{\lambda_{0}}\text{,}$$

where Φ(*x*, *y*) is the phase profile of the device, *h*(*x*, *y*) describes the height of the laser written structure, and *L* is the total thickness of the LC layer.

By ignoring the common phase component and considering only the phase that varies with *h*(*x*, *y*), the expression in Equation (1) can then be simplified to:

(2) $$\Phi \left(x,y\right)=\frac{2\pi \left(n_{\mathrm{ave}}-n_{o}\right)h\left(x,y\right)}{\lambda_{0}}\text{.}$$

Equation (2) can be rearranged to give the desired height profile of the fabricated structure, given the phase profile:

(3) $$h\left(x,y\right)=\frac{\lambda_{0}\Phi \left(x,y\right)}{2\pi \left(n_{\mathrm{ave}}-n_{o}\right)}\text{.}$$

For an optical vortex beam generator, we require a linear phase ramp in *α*, the azimuthal angle around the device such that:

(4) $$\Phi \left(\alpha \right)=l_{\max}\alpha \text{,}$$

where *l*_max_ is the maximum desired vortex order that the device will produce. The height profile of the laser written device can then be written as:

(5) $$h\left(\alpha \right)=\frac{\lambda_{0}l_{\max}\alpha }{2\pi \left(n_{\mathrm{ave}}-n_{o}\right)}\text{.}$$

## 2 Methodology

### 2.1 Sample preparation and device fabrication

The OV devices were fabricated into commercially available glass cells sourced from Instec. The inner surfaces of the substrates of these glass cells were coated with an indium tin oxide (ITO) layer, which acted as the transparent electrodes, that were deposited beneath a polyimide alignment layer. For the top and bottom substrates, the alignment layers were mechanically rubbed along antiparallel directions to promote a planar alignment of the nematic LC in the absence of an applied voltage. Glass spacer beads, with a diameter of 20 μm, were dispersed throughout the device to define the air gap and subsequently the thickness of the LC layer. Before filling, indium solder was used to attach wires to the ITO electrodes and the empty glass cells were then attached to microscope slides using UV-curing glue. A mixture composed of a nematic host LC (79.8 wt% BL006, Merck KGaA), a reactive mesogen (19.2 wt% RM257, Synthon Chemicals), and a photoinitiator (1 wt% IR819, Ciba-Geigy) was capillary filled into the cell at a temperature of 70 °C.

After capillary filling of the LC mixture, two-photon polymerization direct laser writing was performed using a Spectra Physics Mai Tai Ti:Sapphire laser that generated ∼100 fs pulses at a repetition rate of 80 MHz and a center wavelength of 780 nm. The average laser power at the LC layer was 26.2 mW. The laser was focused into the LC cell using a 0.45 NA, 20X objective lens. The LC cell was translated relative to the fixed laser focus using an Aerotech ANT95XY/ANT95V XYZ translation stage. The shutter was left open for the full duration of the fabrication and polymer network was written as a series of concentric helices, with a spacing of 1 μm and a writing speed of 100 μm/s. A function generator was used to apply a 1 kHz 200 Vpp square wave to the cells during the fabrication to ensure a homeotropic alignment of the director at the moment of exposure to the femtosecond laser. The device was designed with a maximum polymerization height of 10.5 μm, which was chosen to generate a second-order OV across the visible spectrum.

### 2.2 Characterization

Polarized optical microscope (POM) images were acquired using a BX51 Olympus microscope with the device positioned between a pair of crossed polarizers, and the rubbing direction (director orientation) at 45° to the transmission axes of the polarizers. The illumination source was filtered to leave a 10 nm band centered at 660 nm to enable inference of the phase profile and to avoid unwanted photopolymerization of the LC mixture after device fabrication. Images were captured using a Retiga R6 Qimaging camera.

POM images can be modeled using Jones calculus, where the LC device can be considered an arbitrary retarder with a spatially varying profile. In this way, it can be shown that the electric field at a given position on the output of the second polarizer is expressed by:

(6) $$E\left(x,y\right)\propto \sin\left(\frac{\gamma \left(x,y\right)}{2}\right)\left(\sin\left(\theta \left(x,y\right)\right)\cos\left(\theta \left(x,y\right)\right)\right)\text{,}$$

where *γ*(*x*, *y*) is the retardance of the sample and *θ*(*x*, *y*) is fast axis orientation.

From Equation (6), we can see that for a device with a fixed fast axis orientation (for example, an LC cell with planar alignment), the transmitted intensity is described by:

(7) I(x,y)∝1−cos(γ(x,y)),

while for a device with a fixed retardance (for example, a q-plate operating in the half wave condition), the transmitted intensity is described by:

(8) I(x,y)∝1−cos(4θ).

**Figure 2:**
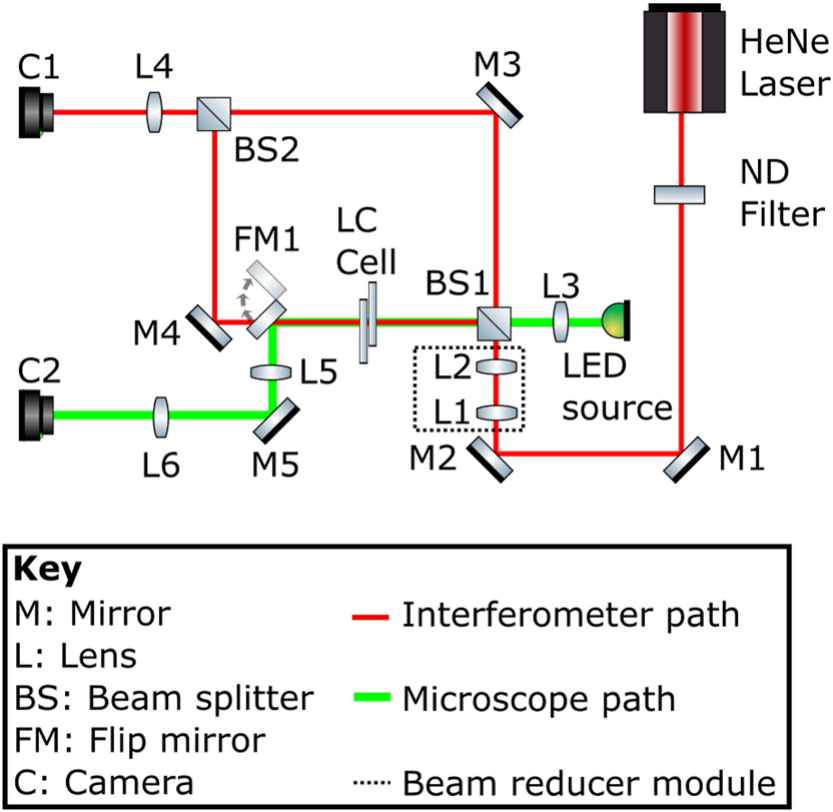
Schematic of the Mach–Zehnder interferometer and imaging system used to characterize the laser-written liquid crystal optical vortex beam generators fabricated in this work. The continuous wave He–Ne laser emitted at a wavelength of *λ* = 633 nm. The liquid crystal optical vortex beam generator is labeled as LC cell in the diagram.

In addition to the POM images, the interferometer presented in the schematic in Figure 2 was used to characterize the LC OV generators. Here, a red He–Ne laser (*λ* = 633 nm) was used as the illumination source and a monochrome CMOS camera was used as the detector. The green beam path shows a separate microscope configuration that was used to align the OV beam generator written into the LC layer with the incident He–Ne laser beam. Far-field images were obtained by blocking the reference arm of the interferometer, and the lens, labeled L4 in the Figure, ensured that the Fourier plane of the light field from the device was projected onto the camera, C1. For the planar interference images, a small misalignment was introduced between the beams coming from the device and reference arms such that they overlapped with a slight angular deviation on the camera. Spherical interference images were subsequently generated by adding a focusing lens into the reference arm to introduce a spherical phase profile onto the reference beam, which was recombined with the OV at the camera. To test the devices at a range of wavelengths, the He–Ne laser was replaced with laser diodes with *λ* = 520 nm and *λ* = 780 nm.

All of the experimental work was carried out in temperature-controlled labs at 22 °C.

### 2.3 Liquid crystal simulations

Simulations of the OV-LC devices were carried out using a minimization of the free-energy as described by continuum theory. A one-constant approximation was used to reduce the Frank free-energy equation to the form

(9) $$F_{\text{d}}=\frac{1}{2}K\left[{\left(\nabla \cdot n\right)}^{2}+{\left(\nabla \times n\right)}^{2}\right]\text{,}$$

where *K* represents the single elastic constant and **n** is the director. By assuming there is no twist present in the director profile, **n** can be expressed in Cartesian coordinates as

(10) $$n=\left(\begin{array}{c} \cos\left(\theta \left(x,y,z\right)\right)\\ 0\\ \sin\left(\theta \left(x,y,z\right)\right) \end{array}\right)\text{ ,}$$

where *θ* is the tilt angle of the director. For our devices, an electric field is applied to the LC layer, which adds the following contribution to the free-energy density

(11) $$F_{\text{E}}=-\frac{1}{2}\Delta \epsilon \epsilon_{0}{\left(E\cdot n\right)}^{2}\text{ ,}$$

where Δ*ϵ* is the dielectric anisotropy at 1 kHz and *ϵ*_0_ is the dielectric permittivity of free-space. As there are no in-plane electric fields, *E* ⋅**n** can be written as *E* sin(*θ*) and so the overall free-energy density equation can be written as

(12) $$F=\frac{1}{2}K\left[{\left(\nabla \cdot n\right)}^{2}+{\left(\nabla \times n\right)}^{2}\right]-\frac{1}{2}\Delta \epsilon \epsilon_{0}{\left(E\sin\left(\theta \right)\right)}^{2}\text{ .}$$

Finally, the resulting Euler–Lagrange equation can then be written as:

(13) $$K\nabla^{2}\theta +\Delta \epsilon \epsilon_{0}\sin\left(\theta \right)\cos\left(\theta \right)E^{2}=0\text{ .}$$

**Figure 3:**
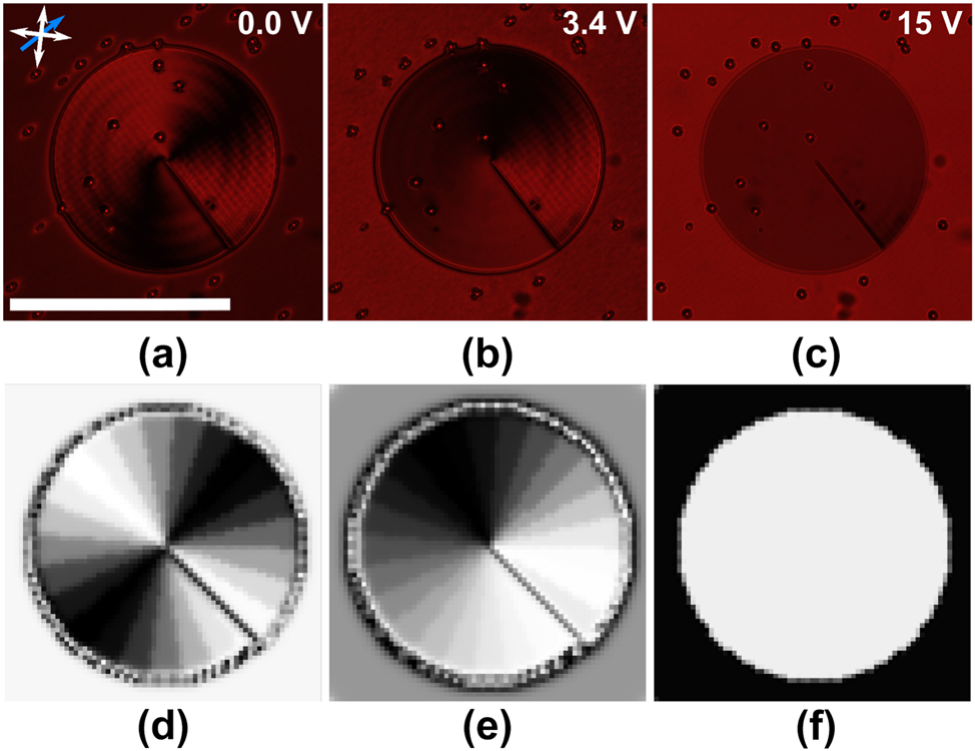
Polarized optical microscope images for the laser written liquid crystal optical vortex beam generator. (a)–(c) Experiment results and (d)–(f) results from simulations. The scale bar in (a) represents a length of 600 μm. The double-headed white arrows show the orientation of the polarizer and analyzer while the blue arrow shows the orientation of the rubbing direction. The voltages applied to the LC layer are shown in the top right corner of each image obtained from experiments. These same voltages were used in the corresponding simulations.

The simulations conducted in this work ran a minimization of the Euler–Lagrange equation twice. The first round of iterations computed the director profile when the writing voltage was applied, taking *θ*(*x*, *y*, *z*) = *θ*_pretilt_ as the initial condition throughout the bulk. The director profile from the first round was then taken as the initial condition for the second round of iterations whereby the minimization was performed to determine the director profile at a given voltage following device fabrication. The polymerization process was modeled by locking the director orientation within the polymerized region. This was achieved as follows: in each iteration, after the director field was updated for the full LC volume, within the volume that was polymerized, the values obtained from the iteration were replaced with the initial condition from the second round. The generated 3D director profile was then used to calculate the retardance profile of the device and a Jones matrix calculation was performed to produce simulated POM images, examples of which are shown in Figure 3(d)–(f).

Initial results from the simulations revealed that a lower voltage was required postfabrication to achieve a given vortex order than that observed experimentally. Within the center of the laser focal volume, a strongly cross-linked rigid polymer network is formed and hence the director is fixed. We suggest that in the surrounding volume, polymer diffusion leads to a fraction of the reactive mesogen cross-linking, forming a weakly cross-linked polymer network, resulting in an increase in the elastic constants of the “nonpolymerized” mixture, but not to a rigid structure. To account for this discrepancy between simulations and experiments, the elastic constant was multiplied by a variable scale factor in the second round of the simulation. The best agreement between experiments and simulations was observed when this scale factor was set to a value of 17. Based on the work in [27], this value seems reasonable.

### 2.4 Laser transmission simulations

Simulations of the far-field images of the intensity profile of the OV beam involved the use of a Fourier transform to calculate the field in the Fourier plane of the lens as

(14) $$U\left(x,y\right)=e^{j\pi \frac{u^{2}+v^{2}}{\lambda f}\left(1-\frac{z}{f}\right)}F\left(U\left(u,v\right)\right)\text{ ,}$$

where *U*(*x*, *y*) is the field in the Fourier plane as a function of coordinates *x* and *y*, *U*(*u*, *v*) is the field object plane as a function of coordinates *u* and *v*, *λ* is the wavelength of the light, *f* is the focal length of the lens, *z* is the distance between the lens and plane in which the image is being formed, and *F*() is the Fourier transform operator. The near field was produced as an ideal helicoidal phase ramp, where the discontinuity was scaled to match the desired vortex order, with a 2-D Gaussian intensity profile.

For the interferometry simulations, the same Fourier transform calculation was run on the reference beam as this also passed through the same relay lens. For the planar interference study, the output from this calculation provided the appropriate focal spot onto which a phase ramp was imposed. Similarly, for the spherical interference simulations, a spherical phase profile was applied to the input field and then the output field was calculated using the Fourier transform. In all cases, the computed output fields were then converted into the intensity profiles shown here.

## 3 Results and discussion

### 3.1 POM imaging

POM images of the manufactured device are shown in Figure (a)–(c). As shown in Equation (6), the transmission intensity in these images indicates the retardance of the device: high intensity corresponds to half-wave retardance, whereas low intensity shows full-wave retardance. The fringes around the device indicate the phase ramp around the device at a given voltage. For voltages equal to and greater than 15 V (Figure 3(c)), there is negligible intensity variation across the device and, therefore, a flat phase profile (*l* = 0). As the voltage starts to decrease, fringes begin to appear and precess around the laser written structure (the OV beam generator), until at 3.4 V (Figure 3(b)), there is a full cycle from light-to-dark-to-light that can be seen, indicating a 2*π* radian phase ramp and, therefore, the condition for generating an OV with *l* = 1. As the voltage is further reduced, another fringe pair appears until at 0 V (Figure 3(a)), two full cycles are visible around the device, indicating a 4*π* radian phase ramp and, therefore, the condition for generating an OV with *l* = 2.

The results for the corresponding simulations at 0 V, 3.4 V, and 15 V are shown in Figure 3(d)–(f), respectively, where the changes in intensity from light to dark are clearly emphasized for the different applied voltages. These simulations demonstrate good qualitative agreement with experiment.

It should be noted that these results differ from those seen for a q-plate of the same vortex order. As described in Equation (8), the transmitted intensity in the POM images varies with 1 − cos(4*θ*), and as discussed previously, for an *n*th order vortex, the fast axis must rotate by *nπ* around the optic axis of the device. Considering both of these factors, we can see that the POM images of a q-plate will exhibit twice as many azimuthal fringes as an HPP of the same order.

### 3.2 Transmission characterization

**Figure 4:**
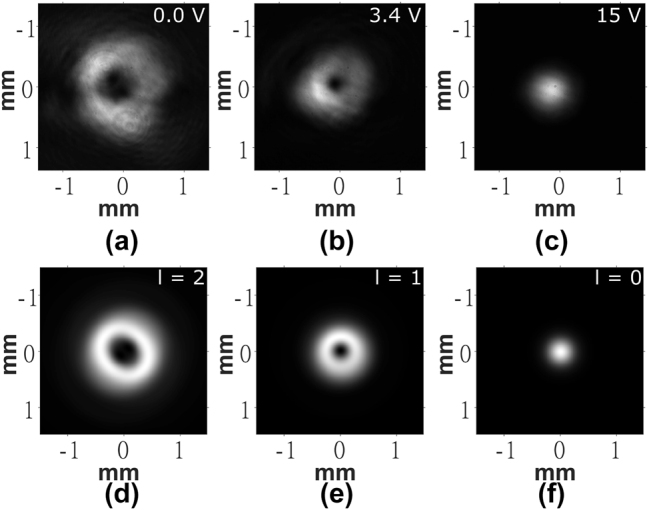
Far-field images of the intensity of the He–Ne laser captured for the laser written liquid crystal optical vortex beam generator. (a)–(c) Experimental results and (d)–(f) results from simulations. The voltages applied to the LC layer are shown in the top right corner of each image obtained from experiments. The order, l, is labeled in the top right corner of each image obtained from simulations.

While the POM images give an impression of the phase profile of the device, they do not yield direct evidence of the OV beam generation. To test the performance of the LC device as an OV generator, additional experiments were conducted. First, a He–Ne laser beam was passed through the device and the far-field intensity pattern was imaged onto a monochrome camera using the optical system illustrated in Figure 2. Figure 4(a)–(c) shows the far-field patterns produced at the voltages previously identified for producing a 2nd, 1st, and 0th order OV, respectively. To accompany the experiments, simulations were carried out using ideal vortex beam phase profiles as the input to the transmission simulations described in Section 2.4. The results for ideal OVs with *l* = 2, *l* = 1, and *l* = 0 are shown in Figure 4(d)–(f), respectively.

**Figure 5:**
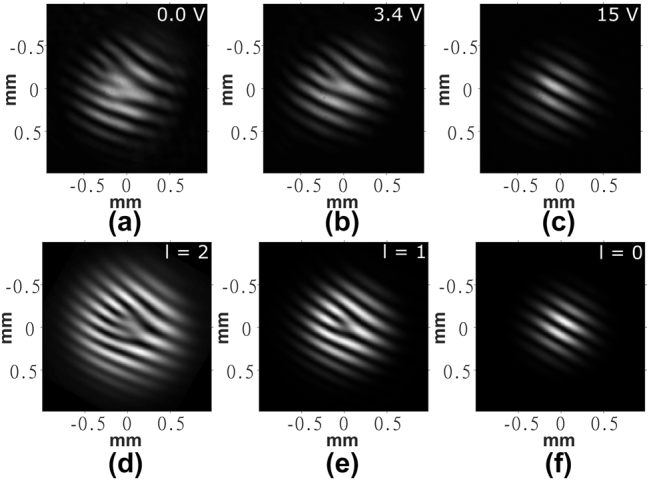
Far-field interferogram images captured for the laser written liquid crystal optical vortex beam generator when a planar wave propagated along the reference arm of the Mach–Zehnder interferometer in Figure 2. (a)–(c) Experiment results and (d)–(f) results from simulations. The voltages applied to the LC layer are shown in the top right corner of each image obtained from experiments. The order, l, is labeled in the top right corner of each image obtained from simulations.

The next step was to determine the OAM of the OVs. For this characterization, the Mach–Zehnder interferometer, discussed in Section 2.2, was used with a plane wave propagating along the reference arm. A small perturbation was also applied between the reference and device arms to create fringes in the far-field interferogram. The resulting interferograms are shown in Figure 5(a)–(c) at the same voltages used previously. When a voltage of 15 V was applied to the LC device (Figure 5(c)), corresponding to *l* = 0, we see linear fringes appear as expected when two off-axis plane waves interfere with one another. However, as the voltage was decreased to generate OVs with *l* = 1 and *l* = 2, the fringes remain but now with one bifurcating, as depicted in Figure 5(b), and one trifurcating, as shown in Figure 5(a). This splitting of the fringes is due to the additional 1 and 2 waves of phase difference on either side of the discontinuities in the first and second-order OV, respectively, hence confirming the magnitude of *l* (OAM) as previously determined from the microscope images. Again, simulations were carried out with the only modification being the addition of an off-axis plane wave to the simulated OV in the far-field to confirm the interferograms. The corresponding results from simulations are shown in Figure 5(d)–(f) and, as before, match the experimental results very well.

**Figure 6:**
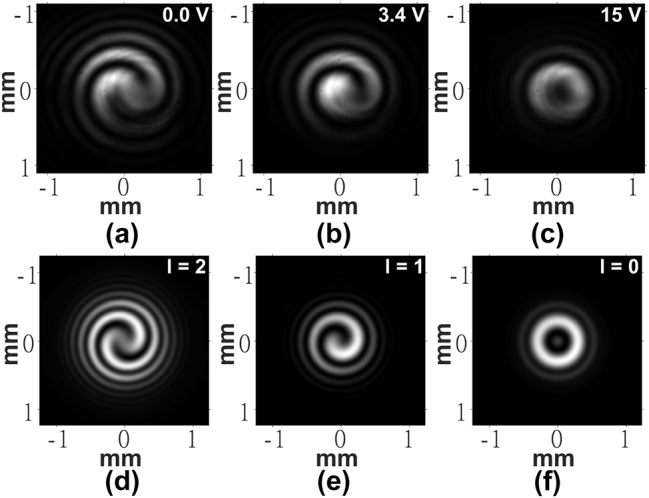
Far-field interferogram intensity images captured for the laser written liquid crystal optical vortex beam generator when a spherical wavefront was added to the reference arm of the Mach–Zehnder interferometer. (a)–(c) Experiment results and (d)–(f) results from simulations. The voltages applied to the LC layer are shown in the top right corner of each image obtained from experiments. The order, *l*, is labeled in the top right-hand corner of each image obtained from simulations.

To test the chirality and confirm the OAM of the LC-OV generator, a weakly focusing lens was introduced into the reference arm of the interferometer to impart a spherical wavefront on the reference beam. The beams were aligned to be paraxial and overlapping at the camera. In this way, the phase profile of the OV can be observed. With an applied voltage of 15 V (Figure 6(c)), when the device produces a 0th order vortex (plane wave), a pair of concentric rings can be seen with the inner ring thicker and brighter than the outer ring. This corresponds to a plane wave interfering with a spherical wave and gives an estimate of the phase depth of the spherical wave as 4*π* rad. Alternatively, when 3.4 V was applied to produce an OV with *l* = 1 (Figure 6(b)), a single-armed anticlockwise spiral can be seen. As the spherical wave has a phase depth of 4*π* radians, the spiral goes through two full rotations, as the phase profile of the OV rotates once per wave. Similarly, in the absence of an applied voltage, corresponding to an OV with *l* = 2 (Figure 6(a)), a two-armed anticlockwise spiral can be seen, with each arm completing two full turns. As expected, there is no change in the chirality of the OV with voltage, as indicated by the spirals in Figure 6(a) and (b) both rotating in the same direction. As before, the corresponding simulation results for OVs with *l* = 2, *l* = 1, and *l* = 0 are shown in Figure 6(d)–(f).

The final characterization step was to test the response of the device at a range of wavelengths. For this, additional far-field measurements were taken with laser sources operating at *λ* = 520 nm and *λ* = 780 nm. For these measurements, the incident laser had a greater beam diameter than that of the LC-OV generator. As a result, the far-field patterns appear as spirals, due to the interference between the optical vortex and the surrounding annular plane-wave. Accounting for this overfilling of the device aperture in the simulations, vortex orders (integer or fractional) produced by the device at different wavelengths and voltages could be estimated. The vortex order generated by the device in experiment was determined by visually registering it with those produced by the corresponding simulation.

**Figure 7:**
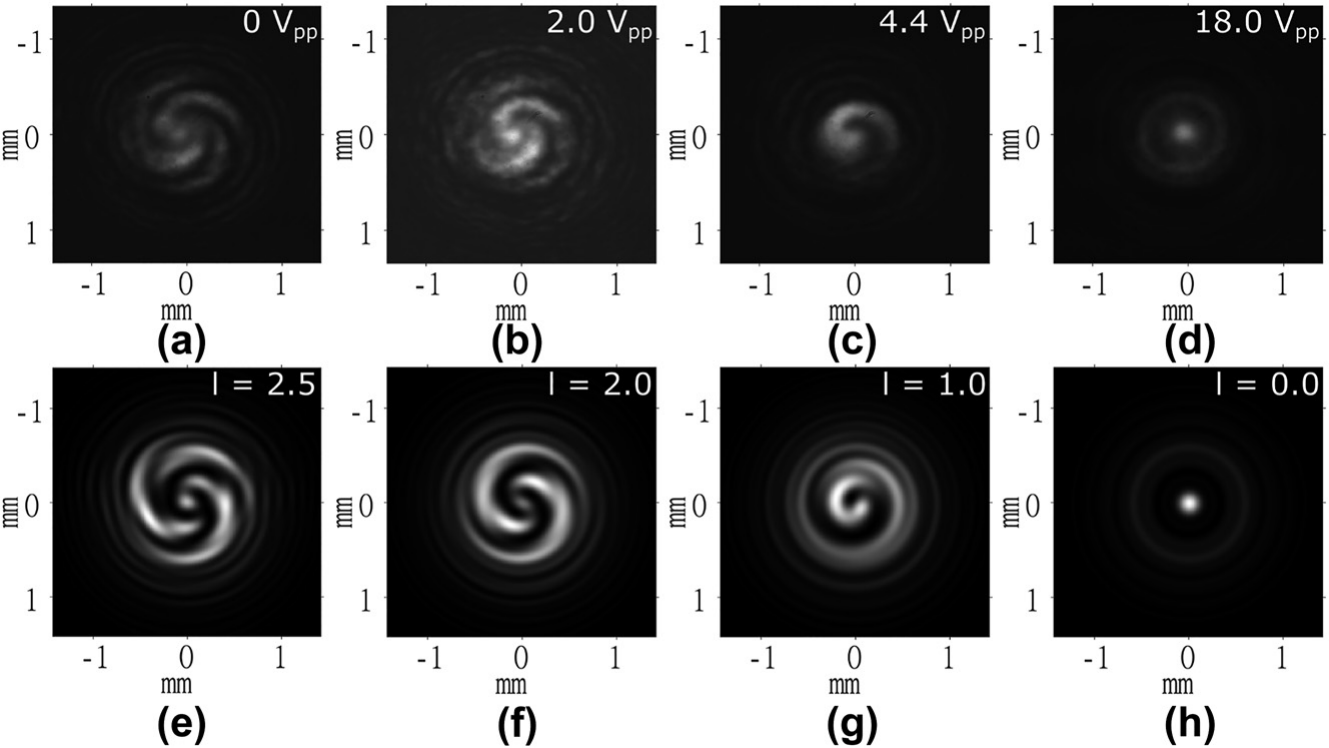
Far-field images of the intensity of the 520 nm diode laser captured for the laser written liquid crystal optical vortex beam generator when the laser beam was wider than the OV generator. (a)–(d) Experiment results and (e)–(h) results from simulations. The voltages applied to the LC layer are shown in the top right corner of each image obtained from experiments. The order, l, is labeled in the top right corner of each image obtained from simulations.

**Figure 8:**
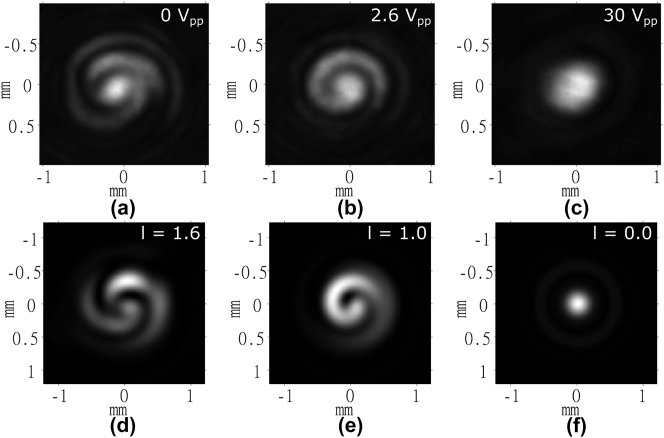
Far-field images of the intensity of the 780 nm diode laser captured for the laser written liquid crystal optical vortex beam generator when the laser beam was wider than the OV generator. (a)–(c) Experiment results and (d)–(f) results from simulations. The voltages applied to the LC layer are shown in the top right corner of each image obtained from experiments. The order, l, is labeled in the top right corner of each image obtained from simulations.

These results are shown in Figures 7 and 8 and show that the device could produce OVs with a maximum order of ∼2.5 at *λ* = 520 nm and ∼1.6 at *λ* = 780 nm. These values correspond to those expected from Equation (5), assuming no significant change to the liquid crystal refractive indices over this wavelength range.

## 4 Conclusions

This paper demonstrates the proof-of-concept and performance of a laser-written liquid crystal (LC) optical vortex generator. By varying the voltage applied to the single electrode pair on the LC cell, the orbital angular momentum of the generated vortex can be varied from 0 to 2 at the design wavelength of *λ* = 633 nm, providing a pathway for tuneable optical vortex generation with very simple drive electronics compared to those needed for driving a spatial light modulator. To achieve tuneability over a greater OAM range, there are several approaches that could be explored for future development. In a single LC device, one approach is to increase the maximum height of the vortex generator that can be fabricated such that a single drive voltage could continuously tune over a greater range of orders. Alternatively, due to the simple drive electronics and transmissive mode of these devices, it would also be possible to stack multiple LC elements together, with different vortex beam generators in each LC layer. Due to the flexibility of the manufacturing laser writing process, we envisage that a stack of devices with each optimized to yield the maximum tuning range: by increasing the gradient of the phase ramp and adding *n* additional 4*π* discontinuities, a similar device could be made that can switch between a 0, an *n*, and a 2*n* optical vortex. Taking *n* as 3, and stacking that device with the one presented here, would enable continuous switching of the OAM from 0 to 8 with 2 electrode pairs. Following the same scaling rule, 4 stacked devices, requiring only 4 drive voltages, would enable continuous OAM tuning from 0 to 80.

These laser-written LC optical vortex beam generators have also been found to be functional at two other wavelengths, *λ* = 520 nm and *λ* = 780 nm, demonstrating polychromatic capability. Due to the use of the photoinitiator IR819, to prevent curing of the nonpolymerized LC, the devices cannot be used where there is a large contribution of UV/blue wavelengths. In the future, optimized mixtures could be developed along with improvements in the laser writing process in order to remove this limitation on the durability of the devices.

Polarizing optical microscope images of the device have been compared with the results from simulations in the context of a minimization of the free-energy using an Euler–Lagrange approach. Good agreement between laser transmission experiments and ideal vortex beam simulations is obtained in terms of both chirality and vortex order. These devices have a broad range of potential applications spanning quantum physics and fundamental studies of fractional optical vortices to optical communications and optical tweezing.
